# Successful treatment of orbital Langerhans cell histiocytosis with stereotactic radiosurgery: A case report and literature review

**DOI:** 10.1002/ccr3.7506

**Published:** 2023-06-19

**Authors:** Emily Etter, Benjamin Bosse, Yi Peng Wang, Susan Hiniker, Justin Oh

**Affiliations:** ^1^ University of Nevada Reno School of Medicine Reno Nevada USA; ^2^ Department of Radiation Oncology Stanford University School of Medicine Stanford California USA

**Keywords:** langerhans cell histiocytosis, orbital, stereotactic radiosurgery

## Abstract

Langerhans cell histiocytosis (LCH) is a rare inflammatory myeloid neoplasm arising from the proliferation of pathologic Langerhans cells. LCH has a spectrum of presentations predominantly affecting male pediatric patients. As LCH is a relatively uncommon diagnosis, there is no standard of care for treatment of the disease and treatment is based largely on clinical judgment, lesion characteristics, and symptoms at presentation. Here we present a case of unifocal, isolated orbital LCH in a 19‐year‐old young man treated initially with surgical resection. Follow‐up imaging 2 months later demonstrated significant regrowth of the mass and no other sites of disease. The recurrent orbital disease was treated with stereotactic radiosurgery (SRS) to 7 Gy in one fraction. Near complete resolution of the mass was achieved with no recurrence after 1.5 years of follow‐up. SRS for treatment of orbital LCH is a novel treatment not previously described in the literature which may provide benefit in select cases.

## Graphical abstract

MRI of the brain demonstrating Langerhans Cell Histiocytosis (LCH) of an orbit.

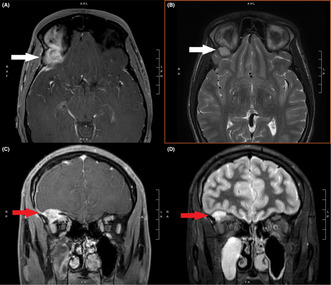

## 1 INTRODUCTION

Langerhans cell histiocytosis (LCH) is a rare inflammatory myeloid neoplasm arising from the proliferation of pathologic Langerhans cells which act as antigen presenting dendritic cells in the epidermis. A Birbeck granule is a unique intracellular organelle finding on electron microscopy that is diagnostic for LCH.^1^, ^2^ LCH mainly affects the pediatric population with an estimated annual incidence of between 2 and 10 cases per million people under the age of 15.^1^, ^3^, ^4^ Though it is more common in children, adult cases are well documented in the literature with evidence of an increased incidence in males over women across all populations.^1^, ^5^, ^6^

Langerhans cell histiocytosis is an umbrella term for a group of diseases previously called Histiocytosis X.^1^, ^2^ LCH can be categorized several different ways: historic reports use two main categories, single system or multisystem LCH.^7^ The current literature categorizes using three main clinical syndromes; localized or unifocal LCH (eosinophilic granuloma), chronic recurring LCH (Hand‐Shullar‐Christian disease), and acute disseminated LCH (Letterer–Siwe disease) which presents as a multisystem disease.^4^ Orbital LCH accounts for approximately 20% of these diagnoses, most commonly with associated intracranial or systemic involvement. There have been few reported cases of unifocal, isolated orbital LCH.^4^

## 2 CASE REPORT

**FIGURE 1:**
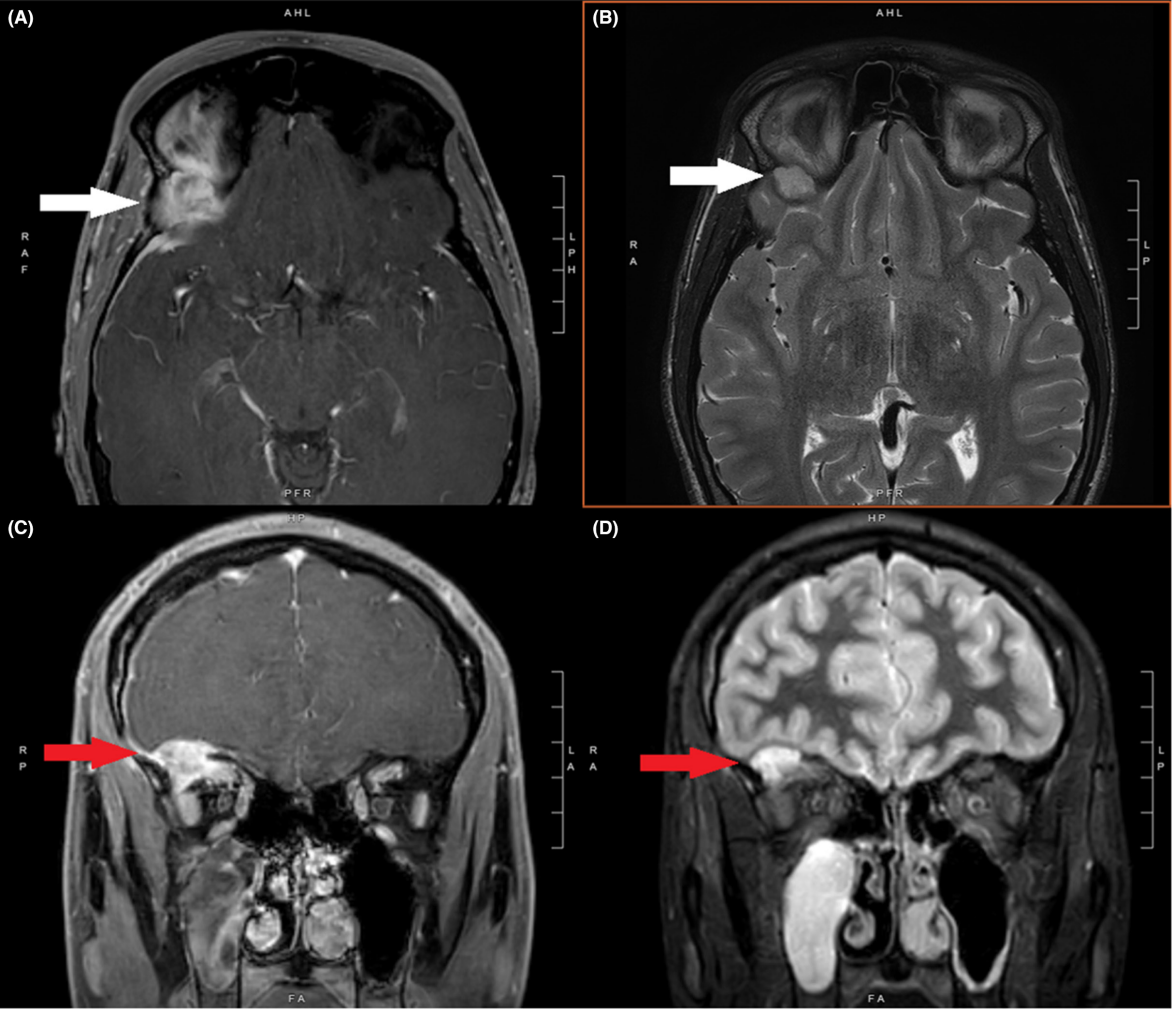
Initial MRI of the brain demonstrating enhancing heterogeneous mass (arrow) in the superior‐lateral aspect of the right orbital roof and extension into the anterior cranial fossa. (A) T1 post‐contrast axial (B) T2 axial (C) T1 post‐contrast coronal (D) T2 coronal.

The patient is a 19‐year‐old male with a past medical history significant for multiple concussions who presented to a family medicine clinic with complaints of persistently worsening right sided frontal headaches starting 2 weeks prior. At the time, he also noted right eye swelling that did not resolve with cold compress or non‐steroidal anti‐inflammatory medication. The headache pain was described as a pounding sensation with no associated symptoms of photophobia, hyperacusis, nausea, epiphora, fevers, or night sweats. The headaches were initially intermittent but occurred daily until 2 days prior to presentation at which point it became constant and debilitating. The patient also noted intense pressure behind the right eye that was worse when he tilted his head backwards. The patient had no significant contributing family history. The patient's vitals and physical exam were normal including a detailed neurological exam demonstrating no focal deficits. Given the history and physical exam findings, differential diagnoses of migraine, tension headaches, cluster headaches, and infection were felt to be less likely than a possible space occupying lesion in the brain. An MRI of the head (Figure 1) demonstrated an enhancing heterogeneous mass in the right superior‐lateral aspect of the right orbital roof with extension into the anterior cranial fossa. This finding prompted admission to the hospital. An extensive blood workup was largely normal including a normal white blood cell count and complete metabolic panel. He was noted to have a slightly elevated absolute monocyte value of 0.58 K/μL (0–0.4 K/μL), absolute eosinophil value of 0.3 K/μL (0–0.2 K/μL), and a prothrombin time of 15.7 s (11.5–14.7 s), INR of 1.3 (0.9–1.2). A full‐body pet scan demonstrated no other lesions or abnormal glucose uptake. The patient was evaluated by neurosurgery and ophthalmology teams and underwent a lateral orbitotomy with stereotactic guidance for excision of the mass and affected bone within the anterior cranial fossa. No intralesional steroids were used due to the proximity of the lesion to the brain. Postoperatively, the patient was given indomethacin for pain, and edema. The pathology report of the excised tissue demonstrated a proliferation of histiocytic tissue with mixed neutrophilic and eosinophilic proliferations. Immunohistochemistry staining was positive for CD1a and langerin confirming a diagnosis of LCH. As this was a unifocal lesion, a more conservative first‐line approach of surgical resection with postoperative indomethacin and close observation was trialed.

**FIGURE 2:**
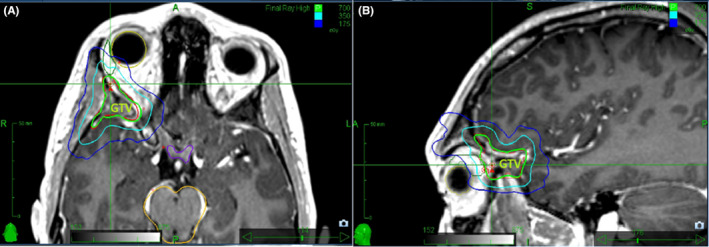
Cyberknife stereotactic radiosurgery plans with gross total volume with isodose lines (Green: 100%, Cyan: 50%, Blue: 25%), prescribed to GTV to cover 95% of the volume at 80% isodose line.

**FIGURE 3:**
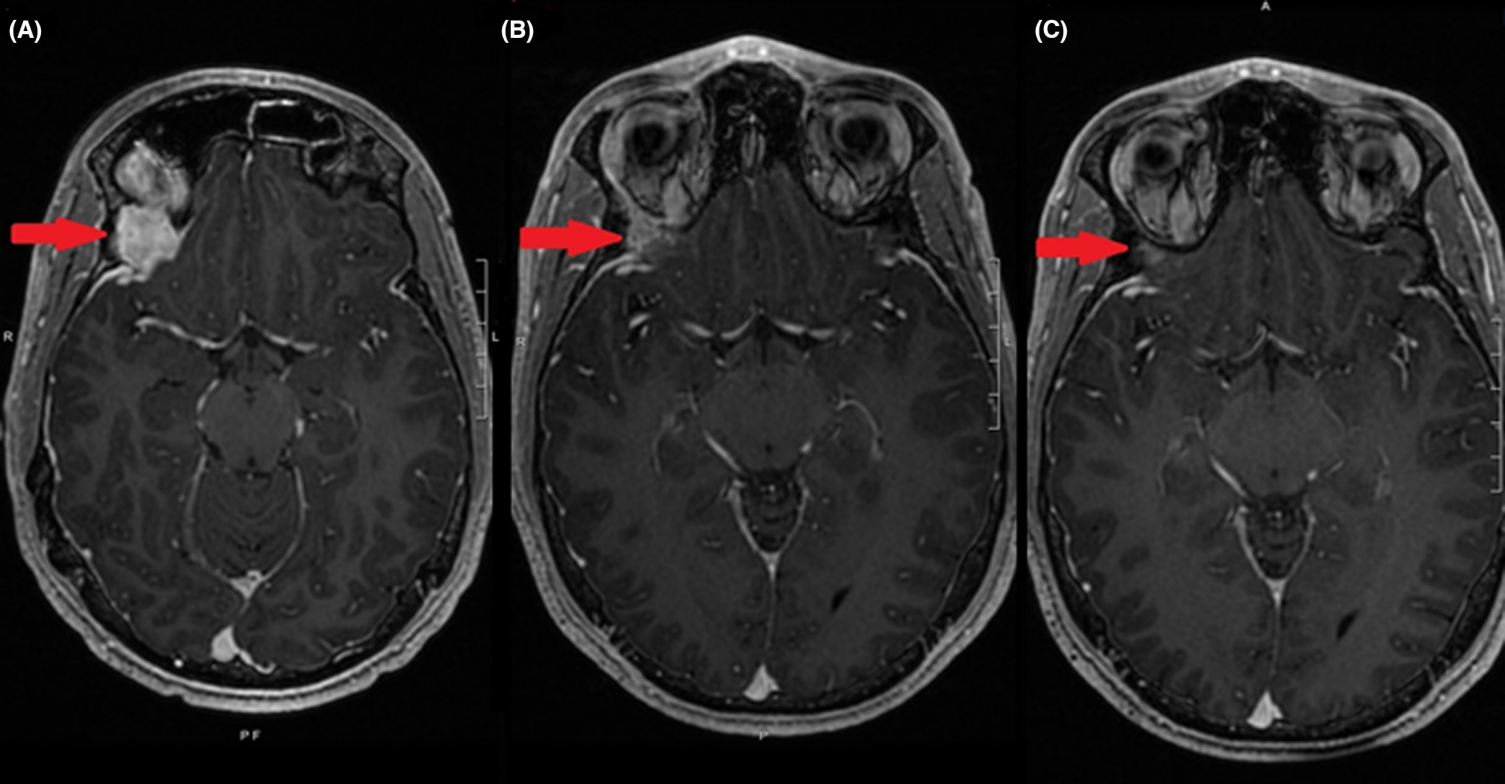
Regression of the right orbital Langerhans cell histiocytosis. Red arrow points to the initial site of disease. (A) MRI brain axial T1 post contrast 2 months after partial surgical resection (B) MRI brain axial T1 post contrast 3 months from radiation (C) Most recent MRI brain axial T1 post contrast, 1.5 years from the radiation.

Follow‐up MRI 2 months after resection showed persistence of the mass indicating residual LCH from a partial resection that was interpreted as having regrown significantly since the time of surgery. Following a patient‐ and family‐centered discussion regarding treatment goals and options, the decision was made to proceed with stereotactic radiosurgery (SRS). Treatment consisted of 7 Gray (Gy) in one fraction prescribed to the gross tumor volume to cover 95% of the volume at 80% isodose line, as outlined in Figure 2. This one‐time radiation treatment was the only treatment provided to the patient following surgical resection. Subsequent imaging demonstrated almost complete resolution of the mass as shown in Figure 3. One and a half years following treatment, there has been no recurrence or complications from the treatment.

## 3 DISCUSSION

Orbital Langerhans cell histiocytosis (LCH) is a rare condition that primarily affect children and adolescences. Here we present a case of a unifocal orbital LCH lesion in a 19‐year‐old male with successful resolution of recurrent LCH after a single 7Gy Cyberknife stereotactic radiosurgery (SRS). To our knowledge, this case report is the first to describe the use of Cyberknife stereotactic radiosurgery for management of unifocal orbital LCH.

Langerhans cell histiocytosis is a proliferation of pathologic Langerhans cells normally found in the body as specialized epidermal dendritic cells. They function to present antigens to other immune cells by traveling through the lymph.^2^ In cases of injury or inflammation, there is evidence demonstrating monocyte‐derived cells from the peripheral blood can migrate to the epidermis and differentiate into Langerhans‐like cells.^2^ As a result, LCH was previously thought of as an immune disease, but a better understanding of the pathogenesis of this disease now lists LCH as an inflammatory myeloid neoplasm.^8^

Langerhans cell histiocytosis predominantly affects children with an annual incidence of 2 to 10 cases per 1 million children under the age of 15, with 90% of the primary lesions occurring in the head and neck.^4^ Although more rare, LCH can also be seen in adults, greater than the age of 18, with an incidence of one to two per 1 million people.^8^ Table 1 highlights case reports of orbital LCH lesions with patients presenting at an average age of 11 years old with the main presenting symptoms of eyelid edema or ptosis. A comprehensive study of 90 patients diagnosed with LCH at the Hematology Service of National Cancer Institute found that LCH was two times more common in pediatric patients compared to adult patients.^23^ Craniofacial LCH presentations are more common in the pediatric population with most craniofacial lesions occurring in the orbit. Maia et al. demonstrated that approximately 35% of pediatric patients diagnosed with LCH presented with orbital lesions compared to only 3% of adult LCH presentations. From systematic retrospective reviews of the literature looking at LCH presentations in the head and neck, Bezdjian et al. and Xu et al. found orbital LCH lesions to account for 24% and 22% of confirmed head and neck LCH cases.^6^, ^24^ These orbital LCH lesions are not associated with increased risk of developing CNS lesions.^1^

**TABLE 1:** Case reports with orbital lesions of Langerhans cell histiocytosis.

Source	*N*	Mean Age (years)	Presenting complaint (*N*)	Treatment (*N*)	Main location (*N*)	Distant metastasis (*N*)	Status at last follow‐up (*N*)	Follow‐up length after treatment
Koka et al.^1^	9	10.12	Eyelid edema (4) proptosis (5)	Surgery/biopsy + OCT	Superior orbital rim (5)	N/A	N/A	1.3 years
Harris and Woo^9^	7	8.14	Eyelid edema (6)	Surgery/biopsy (7) + steroids (4) + radiation (2) (400 and 800 cGy)	Unilateral orbit	None	No recurrence	1 year
Cheung et al.^10^	3	27	Headache and eyelid edema	Surgery/biopsy (3) + radiation (30Gy) (1) + steroids(1)	Unilateral orbit	None	No recurrence	2.1 years
Das et al.^11^	3	6	Periorbital edema and proptosis	Surgery + radiation (15 Gy),	Unilateral orbit (2), Bilateral orbital bone (1)	Spleen (1), facial skin (1)	No recurrence	4.5 years
				Surgery + radiation (10 Gy), Chemotherapy + steroids				
Herwig et al.^12^	5	4.35	Continued swelling after minor trauma	IVCT+ steroids (2), OTC + steroids (1), steroids alone (1)	Unilateral orbit	Bone lesions of leg and skull (1)	No recurrence	5.3 years
Esmaili and Harris^13^	6	2.8	Unilateral periorbital edema	Biopsy/surgery + IL steroids (5) + OCT (4) + 2Cda (2)	Unilateral Orbit	Skin + bone (4), liver (1), lungs (2), bone marrow (1)	No recurrence (5) + one still in treatment after 90 months	3.8 years
Singh et al.^14^	8	8.4	Periorbital edema (6), proptosis (2)	Surgery/biopsy (7), FNA (1) + ILS (8) + IVCT	Unilateral, Superior Orbit	None	No recurrence (7), still in therapy (1)	2.5 years
Kiratli et al.^15^	17	10.7	Proptosis (8), Upper Eyelid Edema (4)	Surgery (17) + steroids (3) + OCT (10)	Unilateral (16), bilateral (1)	Calvarium, femur, facial, temporal, and parietal bones (5)	No recurrence (90%)	4.5 years
Plemel et al.^16^	38	28.19	Upper Eyelid Mass (13), Edema	OCT (7), surgical excision (20), radiation (5), steroids (3), no treatment (1)	Unilateral orbit (34), bilateral (4)	Multisystem (7), lymph node involvement (2), none (29)	No recurrence (22), disease progression or recurrence (6), died (2)	N/A
Narayan et al.^17^	1	16	Diplopia and headache	Surgery	Superior oblique muscle	None	No recurrence	1.3 years
Chang et al.^18^	1	2	Trauma with prolonged lower eyelid edema and discoloration	Surgery + OCT + IVCT	Greater sphenoidal wing, the supraorbital wall, frontal area	Nuchal and inguinal lymph nodes	Still in treatment	N/A
Das et al.^19^	1	28	Diplopia, proptosis, edema	Patient declined treatment	Posterior lateral right orbit	None	N/A	None
Karki et al.^20^	2	6	Trauma with prolonged headaches (1), trauma with incidental lymph node edema (1)	Surgery and steroids (1) surgery alone (1)	Orbit and skull	Skull lesions	No recurrence (1), continuous daily low dose steroids (1)	5 months, 7 years
Gokmen et al.^21^	1	6	Proptosis and eye pain	Surgical excision	Unilateral orbit	None	No recurrence	0.5 years
Alkatan et al.^22^	8	3.8	Eyelid edema, proptosis	N/A	Unilateral orbit (8)	None (6), brain (1), bone marrow (1)	N/A	N/A

*Note*: A comprehensive review of case presentations, diagnosis, treatment, and recurrence are highlighted below. Literature review in PubMed yielded 68 results using the terms “langerhans cell histiocytosis and orbit” of which there are 15 applicable results outlined below. There were no complications with treatment.^1^, ^9^, ^10^, ^11^, ^12^, ^13^, ^14^, ^15^, ^16^, ^17^, ^18^, ^19^, ^20^, ^21^, ^22^

Abbreviations: FNA, fine needle aspiration; ILS, intralesional steroids; IVCT, IV chemotherapy (etoposide, cisplatin), steroids (methylprednisolone or prednisolone); OCT, oral chemotherapy (6 mercaptopurine, vincristine, vinblastine).

A diagnosis of LCH can be made through immunohistochemistry or electron microscopy. The presence of langerin (CD207+) or cluster of differentiation 1a (CD1a) on immunohistochemistry or Birbeck granules on electron microscopy are specific for a definitive diagnosis of LCH.^1^, ^6^ The presence of S‐100, adenosine deaminase, alpha‐mannosidase, and peanut lectin binding are also common markers and two or more of these markers meet criteria from the International Histiocyte Society for a diagnosis of LCH.^1^ Langerhans cell histiocytosis preferentially affects the bone, skin, lungs, and pituitary gland and can have variable presentation.^14^ The extent of system involvement determines a diagnosis of a localized, unifocal eosinophilic granulomas, chronic recurring, or acute disseminate multisystem disease.^4^, ^7^ A diagnosis of high‐risk LCH occurs when pathologic LCH cells are found in the spleen, liver or bone marrow and are associated with a worse prognosis and higher rates of death.^2^

Currently there is no standardized treatment for LCH. Treatment approach is individualized based on clinical presentation. A unifocal lesion affecting the skeletal system is the most common form of LCH.^7^ These lesions can spontaneously regress and no further treatment may be required. Arceci et al. suggests a conservative first‐line treatment of a 2‐ to 4‐week course of oral nonsteroidal anti‐inflammatories such as Naprosyn, indomethacin, or ibuprofen to reduce symptoms and accelerate spontaneous healing by reducing the production of local prostaglandins and interleukins such as IL‐1.^25^ If the lesion does not spontaneously regress, resection or simple curettage is generally first‐line treatment.^3^ For large bone lesions >5 cm radical excision is not indicated due to causing larger and permanent deformities.^7^ Most pediatric patients with a unifocal bone lesion are managed by resection with an intralesional injection of methylprednisolone followed by observation and chemotherapy, with vinblastine being preferred over chlorambucil and etoposide.^6^, ^25^ To minimize risk of long‐term complications, intralesional steroids are typically not recommended for an orbital lesion due to its central location.^25^ Additionally, recent reports suggest that intralesional steroids may lead to an increased rate of recurrence.^4^ More recent literature has shown that a BRAF V600E mutation is found in over 50% of confirmed LCH cases. In this setting, vemurafenib has been efficacious for a number of cases.^8^

If there are doubts about the completeness of the resection or if there is recurrence, radiation at a relatively low dose of approximately 4–8 Gy is suggested for unifocal single system LCH following surgical resection. Radiation is generally more widely used in the adult population as compared to the pediatric population due to possible side effects.^1^, ^8^, ^25^ For orbital tumors, complications related to radiation may include dermatitis, hair loss, early‐onset cataracts, chronic dry or painful eye, optic nerve damage and secondary malignancy; however, due to the low dose used to treat LCH, these risks are very low.^4^ All cases reviewed in Tables 1 and 2 had no complications to treatment discussed in their cases.

**TABLE 2:** Langerhans cell histiocytosis lesions treated with Gamma or Cyberknife radiation.

Source	*N*	Age (years)	Lesion location	Type of SRS	Additional treatment	Dose	Fractions	Prescription isodose	Outcome	Follow‐up length
Faramand et al.^26^	1	2	Skin: disseminated	N/A	Chemotherapy (not specified)	N/A	N/A	N/A	‐	‐
		12	Hypothalamus	N/A	Surgical resection and radiation therapy	N/A	N/A	N/A	‐	‐
		27	Right cerebellum and hypothalamus	Gamma Knife	Surgical excision, corticosteroids, pentoxyphylline	13, max 26 Gy	1f	N/A	‐	‐
		35	Left frontal lobe, right cerebellum	Gamma Knife	Intralesional corticosteroids	14, max 28 Gy	1f	N/A	‐	‐
		39	Regrowth of frontal lobe and cerebellum	Gamma Knife	N/A	12–13 Gy	1f	50%	Stable	3 years
Hong et al.^27^	3	35	Posterior pituitary	Cyber Knife	Craniotomy and Biopsy	18 Gy	3f	80%	Reduced size	7 months
		49	Posterior pituitary	Cyber Knife	Transsphenoidal biopsy	8 Gy	1f	80%	Stable	3 years
		54	Pituitary stalk	Cyber Knife	Transsphenoidal biopsy	10 Gy	1f	80%	Stable	2 years
Cagli et al.^28^	1	24	Left temporal bone and pone	Gamma Knife	Thoracoscopic resection, radiation therapy (30 Gy)	N/A	1f	N/A	Stable	4 years
Tan et al.^29^	1	49	Posterior pituitary	Gamma Knife	Excisional surgery and chemotherapy	N/A	1f	N/A	Increased size after 1 year	1 year
Rio et al.^30^	1	30	Left petrous bone	Gamma Knife	N/A	10, max 11.76 Gy	1f	85%	Stable	2 years

*Note*: Literature review of PubMed using terms “langerhans cell histiocytosis and stereotactic radiosurgery” yielded 11 results with five applicable results highlighting lesions and type of treatment below.^26^, ^27^, ^28^, ^29^, ^30^

Though not generally thought of as a first‐line treatment for LCH, stereotactic radiosurgery (SRS) has been used as first‐line treatment for neurological LCH lesions in several reports that have shown regression and stable tumor outcomes, outlined in Table 2. SRS therapy is a very precise form of radiation most often used for CNS tumors. Gamma Knife (GK) is the oldest and most popular form of SRS worldwide. Gamma Knife uses hundreds of concentrically placed Cobalt‐60 energy beams originating from multiple areas. In comparison, Cyberknife (CK) SRS uses 6 MV photon beams projected from a single robotic arm using a compact linear accelerator. CK uses image‐guided radiotherapy to track objects in time and space allowing slightly more freedom of movement during treatment.^31^

There are currently nine case reports that present patients who have been treated with either GK or CK SRS at some point in their treatment course. There are seven patients with confirmed CNS LCH lesions who had SRS as part of their treatment course demonstrating resolution and stability of their tumors. None of these patients had SRS as their first or only form of treatment for their lesion. The other two patients treated with SRS had Rosai–Dorfman disease, a histiocyte infiltration into the lymph. Hadjipayanis et al. describes a case of a 52‐year‐old male with lesions in the left petroclival area extending into the cavernous sinus. This was treated with gamma knife SRS with complete resolution at a 13 month follow‐up.^32^ Sato et al. describes a case report of a 57‐year‐old with lesions located in multiple areas of the brain including the suprasellar region, left frontal convexity and the cerebellopontine angle. Her lesions were treated with 8–14 Gy gamma knife radiation leading to complete resolution 3 months after treatment with no recurrence at a 3 year follow‐up.^33^

For LCH‐specific lesions, Hong et al. reports three adult presentations of acquired diabetes insipidus which was later found to be caused by a posterior pituitary LCH lesion.^27^ These three cases are the only reports of LCH lesions treated with Cyber Knife radiation and all had tumor stabilization with no recurrence.^27^ The current case report adds to the existing literature exploring the feasibility, effectiveness, and safety of SRS for unifocal LCH and further prospective studies may help to clarify the role of SRS in unifocal LCH.

## AUTHOR CONTRIBUTIONS

**Emily Etter:** Conceptualization; data curation; formal analysis; investigation; methodology; writing – original draft; writing – review and editing. **Ben Bosse:** Conceptualization; data curation; writing – original draft; writing – review and editing. **Yi Peng Wang:** Data curation; investigation. **Susan Hiniker:** Conceptualization; formal analysis; supervision. **Justin Oh:** Conceptualization; data curation; supervision; writing – review and editing.

## FUNDING INFORMATION

No funding was received for this study.

## CONFLICT OF INTEREST STATEMENT

None of the authors have conflict of interest to declare.

## CONSENT

Written informed consent was obtained from the patient to publish this report in accordance with the journal's patient consent policy.

## Data Availability

The data that support the findings of this study are available on request from the corresponding author. The data are not publicly available due to privacy or ethical restrictions.
